# Improving Resilience, Optimism, Purpose, and Loneliness Among Older Adults

**DOI:** 10.1093/geroni/igab046.690

**Published:** 2021-12-17

**Authors:** Rifky Tkatch, Lizi Wu, Laurie Albright, Rachel Ungar, Catherine Zaidel, Yan Cheng, James Schaeffer, Charlotte Yeh

**Affiliations:** 1 UnitedHealth Group, Oak Park, Michigan, United States; 2 UnitedHealth Group, Minnetonka, Minnesota, United States; 3 UnitedHealth Group, Minneapolis, Minnesota, United States; 4 OptumLabs, Minnetonka, Minnesota, United States; 5 AARP Services, Inc., Washington, District of Columbia, United States

## Abstract

Aging Strong 2020 was developed to promote health and well-being and increase resilience by focusing on the pillars of enhanced purpose in life, social connectedness, and optimism. A series of eight interventions over three years tested the feasibility of enhancing these pillars. Interventions included: 1) An expressive writing program, 2) Animatronic pets, 3) A telephonic reminiscent program, 4) An online self-compassion mindfulness program, 5) A technology-based behavior change tool, 6) An online and workbook tool for purpose, 7) An online happiness program, and 8) A peer-to-peer support program. Each program demonstrated efficacy dependent on the pillar targeted and the population sampled. Overall, some improvement was found among participants in resilience (47%), purpose (49%), optimism (44%), and loneliness (48%). Further, participant satisfaction improved in each program with Net Promoter Scores increasing between 7-19 points. Results demonstrate that Aging Strong 2020 was successful, contributing to a holistic model of healthy aging.

